# Serum cross‐linked N‐telopeptide of type I collagen as a potential diagnostic marker for bone metastasis in lung cancer: An updated meta‐analysis

**DOI:** 10.1111/1759-7714.14808

**Published:** 2023-01-30

**Authors:** Haiyan Wang, Mingsheng Hong, Ling Ding, Baona Song

**Affiliations:** ^1^ Deaprtment of Respiratory Hangzhou Third People's Hospital Hangzhou People's Republic of China; ^2^ Department of Ophthalmology Zhejiang Hospital Hangzhou People's Republic of China; ^3^ Department of Gerontology Hangzhou Third People's Hospital Hangzhou People's Republic of China

**Keywords:** bone metastasis, lung cancer, meta‐analysis, NTx

## Abstract

**Objective:**

To investigate the clinical value of serum type I collagen cross‐linked amino terminal peptide (NTx) for the diagnosis of bone metastasis in lung cancer patients by evidence‐based medicine.

**Methods:**

Diagnostic studies relevant to NTx as a serum marker for the diagnosis of bone metastasis in lung cancer patients included in the China National Knowledge Infrastructure, Wanfang, PubMed, Embase, and VIP database from the establishment of the databases to November 2022 were retrieved. Meta‐analysis was conducted with Stata 16.0 software to calculate the combined sensitivity (SEN), specificity (SPN), positive likelihood ratio (+LR), negative likelihood ratio (−LR), diagnostic odds ratio (DOR), and its 95% confidence interval (CI). The total subject working characteristic curve (summary receiver operating characteristic (SROC) curve) was drawn, and the area under the curve was calculated to evaluate its diagnostic value.

**Results:**

A total of 1742 patients with lung cancer were included in 14 articles. Meta‐analysis showed heterogeneity among the studies. The results of random‐effect model analysis demonstrated that the combined SEN, SPN, +LR, −LR, and DOR were 0.76 (95% CI 0.66–0.83), 0.80 (95% CI 0.74–0.85), 3.80 (95% CI 2.90–4.80), 0.30 (95% CI 0.22–0.42), and 12 (95% CI 8–19), respectively, and the area under SROC was 0.85.

**Conclusion:**

Serum NTx has a high clinical value in the diagnosis of bone metastasis in lung cancer patients and can be used as an effective complementary means for imaging diagnosis of bone metastasis in lung cancer patients.

## Graphical abstract

The Fagan diagram shows that when the pretest probability of bone metastasis in lung cancer patients is 70% by imaging inspection, and the results of serum cross‐linked N‐telopeptide of type I collagen (NTx) detection are consistent, the post‐test probability can be increased to 90%; otherwise, the post‐test probability is 42%, which suggests that the combination of serum NTx and imaging inspection can accurately determine the degree of bone metastasis in lung cancer patients.

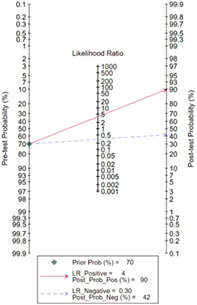

## INTRODUCTION

Lung cancer is one of the most common malignant tumors. According to the latest published cancer epidemiology study, the number of new cases of lung cancer will reach more than 2.2 million in 2020. In addition, lung cancer is the main cause of cancer‐related deaths, with about 1.8 million people dying due to the disease, accounting for 18% of the total number.^1^ Most lung cancer patients are in advanced stages at the time of diagnosis. Bone is one of the common sites of lung cancer metastasis, with 35–40% of lung cancer presenting bone metastasis.^2^ Bone metastases often cause severe bone pain and a variety of complications, including pathological fractures and spinal cord compression syndrome; radiotherapy for pain relief, bone surgery, and hypercalcemia are needed for the prevention and treatment of pathological fractures or spinal cord compression. The complications are collectively referred to as skeletal‐related events (SREs), which not only reduce the quality of life of patients but also shorten their survival period, therefore early diagnosis of bone metastasis is significant for the improvement of the quality of life of patients.

The cross‐linked N‐telopeptide of type I collagen (NTx), a bone metabolism marker in serum, is closely related to bone metastasis in lung cancer patients.^3^ NTx is an important collagen degradation product released by osteoclasts during bone degradation, and it has a high bone specificity (SPN). When bone metastasis occurs, its level usually increases.^3^, ^4^ Urine NTx is one of the important indicators of diagnosis of bone metastasis and evaluation of the prognosis of lung cancer patients.^5^, ^6^ However, conclusions regarding the diagnostic value of serum NTx for bone metastasis of lung cancer patients are different, and the study sample size is small, with little clinical significance. This study evaluated the diagnostic value of serum NTx in patients with lung cancer with bone metastasis through the meta‐analysis of published literatures at home and abroad.

## MATERIALS AND METHODS

A literature search was carried out on the China National Knowledge Infrastructure, Wanfang, PubMed, Embase, and VIP databases, and relevant domestic and foreign studies relevant to serum NTx for the diagnosis of bone metastasis of lung cancer patients published before November 2022 were retrieved. In addition, research references were also manually retrieved. Key words included “cross‐linked N‐telopeptide of type I collagen,” “NTx,” “lung cancer,” “bone metastasis,” “diagnostic,” or “prognosis.” The retrieval strategy adopted the principle of combining subject words with free words. The electronic search languages were English and Chinese.

The inclusion criteria for the literature were as follows: (1) published research on serum NTx for the diagnosis of bone metastasis in lung cancer patients; (2) patients diagnosed with lung cancer by histopathology and at least one bone metastasis or characteristic bone destruction confirmed by imaging diagnosis; (3) enzyme‐linked immunosorbent assay (ELISA) as the detection method of serum NTx; (4) sufficient information to extract or calculate the number of true‐positive (TP), true‐negative (TN), false‐positive (FP), and false‐negative (FN) cases. The exclusion criteria were as follows: (1) overview, lectures, and case reports; (2) literature with incomplete original data or insufficient information to calculate the true positive, false positive, false negative, or true negative; (3) lung cancer without pathological diagnosis.

Data extraction and quality evaluation were conducted by two researchers who independently screened literature and extracted data. Any dispute that arose during the extraction process was resolved through discussion or by soliciting the opinion of a third researcher. The extracted information included the first author, year of publication, country, age, sample size, detection method, and cut‐off value of serum NTx. The true‐positive, false‐positive, false‐negative, and true‐negative data were extracted from each included studies and made cross checking. The quality evaluation of the methodology included in the research adopted the quality assessment of diagnostic accuracy studies (QUADAS) tool.^7^ The evaluation tool comprised 14 items in four parts, including case selection, trials to be evaluated, gold standard, and case flow and progress.

Stata 16.0 software was used for statistical analysis of the included studies. The heterogeneity of the included studies was assessed by *I* ^2^ test and *p* value. When *p* ≤ 0.1 or *I* ^2^ ≥ 50%, heterogeneity was considered to exist and a random‐effects model was used. Otherwise, a fixed‐effects model was applied. Diagnostic sensitivity and specificity were calculated by the equations sensitivity = true positive/(true positive + false negative) and specificity = true negative/(true negative + false positive). The combined sensitivity (SEN), SPN, positive likelihood ratio (+LR), negative likelihood ratio (−LR), diagnostic odds ratio (DOR), and 95% confidence interval (CI) were calculated using a bivariate mixed‐effect model. In addition, the summary receiver operating characteristics (SROC) curve was drawn to estimate the overall diagnostic accuracy of the test. Meta regression analysis was used to find potential factors that may cause heterogeneity, and subgroup analysis was conducted. Deek's funnel plot was illustrated to determine publication bias.

## RESULTS

**FIGURE 1:**
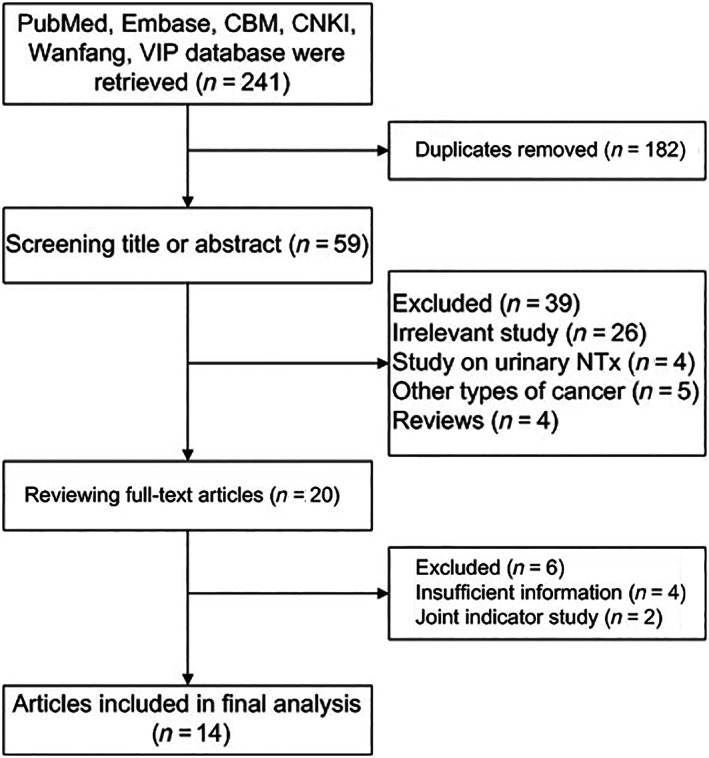
Flow chart for study search

A total of 241 relevant literature sources were preliminarily retrieved from the search results and included for further eavluation. Meanwhile, 221 irrelevant articles were excluded, and 20 were initially included in this study after duplicate checking and reading of titles and abstracts. After careful reading of the full text, six articles that could not be used to obtain the four‐grid data were excluded, and 14 were finally included.^8^, ^9^, ^10^, ^11^, ^12^, ^13^, ^14^, ^15^, ^16^, ^17^, ^18^, ^19^, ^20^, ^21^ Figure 1 shows the document screening process.

Basic characteristic and quality evaluation of the included studies was performed. The 14 articles included in this study involved 1742 patients with lung cancer. Ten studies were from China, and the other four were from Japan, Turkey, and Italy. The sample sizes were in the range of 35 to 312. ELISA was used as the test method for NTx. The average score of QUADAS for the included studies was 10.43 (ranging from 9 to 12), which indicates a good overall quality level. Tables 1 and 2 show the basic characteristics and relevant indicators of the included articles.

**TABLE 1:** General characteristics of included studies

Author	Year	Country	Age Mean/median		NTx		QUADAS	Test method
					Case	Control		
Wang W	2008	China	58.4 (median)	05	24.06 ± 10.67	13.16 ± 9.52	9	ELISA
Zhang SQ	2011	China	39–73	06	25.36 ± 11.07	12.16 ± 7.62	11	ELISA
Lumachi F	2011	Italy	63 (median)	5	33.5 ± 7.2	25.6 ± 3.1	9	ELISA
Zhou ZJ	2011	China	59.93 ± 11.67	8	214.08 ± 262.45	80.38 ± 40.96	12	ELISA
Tamiya M	2012	Japan	68 (23–85)	76	29.4	17.3	10	ELISA
Bayrak SB	2012	Turkey	64.07 ± 8.7	65	22.69 ± 7.98	18.67 ± 6.85	10	ELISA
Tamiya M	2013	Japan	66 (25–86)	50	93.2 ± 105.1	51.6 ± 26.8	11	ELISA
Sun H	2013	China	NA	100	46.18 ± 24.22	23.99 ± 9.05	9	ELISA
Wu Q	2018	China	64 (median)	126	NA	NA	10	ELISA
Zhuang XJ	2018	China	NA	107	47.8 ± 25.3	22.9 ± 9.1	11	ELISA
Cui ZJ	2020	China	59.26 ± 9.98	312	50.79 ± 12.09	8.12 ± 3.99	10	ELISA
Zhao HX	2020	China	61.32 ± 8.86	74	18.59 ± 17.53	9.72 ± 6.09	11	ELISA
Gu LY	2020	China	63 (40–78)	100	NA	NA	12	ELISA
Ma HY	2021	China	53.36 ± 6.77	208	25.46 ± 3.87	13.12 ± 2.03	11	ELISA

Abbreviations: ELISA, enzyme‐linked immunosorbent assays; NA, unavailable; QUADAS, quality assessment for studies of diagnostic accuracy.

**TABLE 2:** Summary of results of NTx in included articles

Author	Year	Cut‐off	TP	FP	FN	TN
Wang W	2008	NA	45	18	5	37
Zhang SQ	2011	NA	55	7	6	38
Lumachi F	2011	30 nM	9	2	7	17
Zhou ZJ	2011	80.38 nM	37	10	4	27
Tamiya M	2012	22 nM	45	14	28	89
Bayrak SB	2012	25.69 nM	10	4	13	38
Tamiya M	2013	22 nM	20	13	30	87
Sun H	2013	26.75 nM	40	11	13	36
Wu Q	2018	21.5 nM	28	13	15	70
Zhuang XJ	2018	31.7 nM	37	5	5	60
Cui ZJ	2020	21.5 nM	139	30	23	120
Zhao HX	2020	8.88 nM	26	13	11	24
Gu LY	2020	0.094 ng/ml	43	20	7	30
Ma HY	2021	17.14 nM	79	36	29	64

**FIGURE 2:**
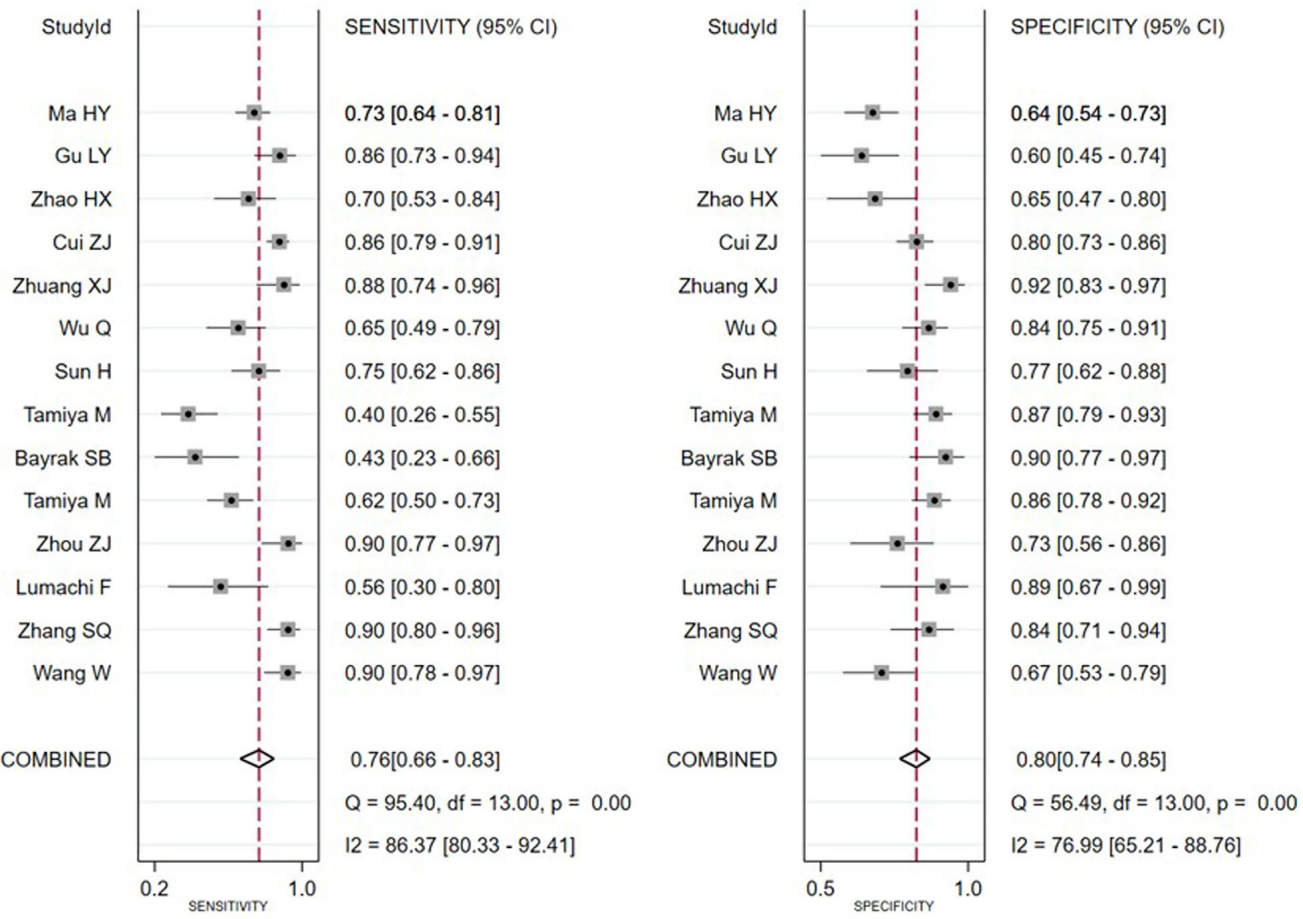
Forest plot of sensitivity and specificity for bone metastases disease detection by serum NTx

**FIGURE 3:**
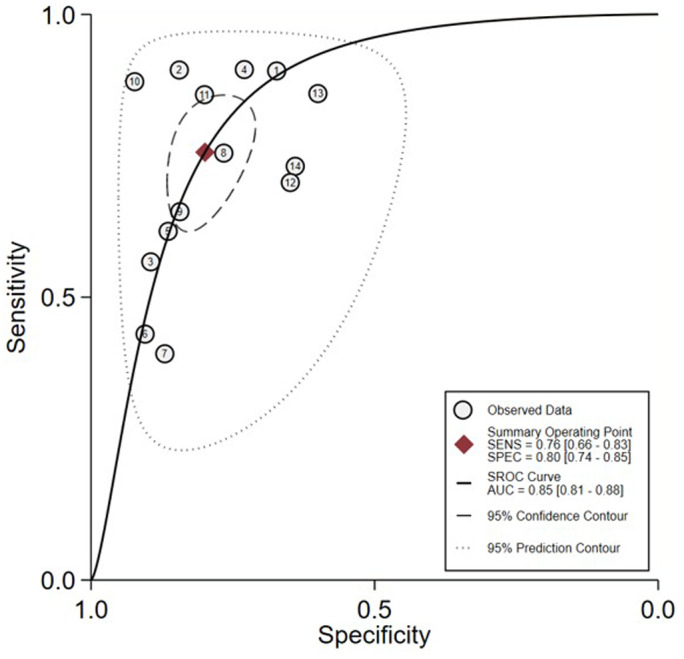
AUC of the ROC curve for bone metastases disease detection by serum NTx

Meta analysis heterogeneity test results showed that *I* ^2^ = 97% and *p* < 0.01, which suggest a possible heterogeneity among the studies, therefore a random‐effect model was used for meta‐analysis. The results showed that the combined SEN, SPN, +LR, −LR, and ODR were 0.76 (95% CI 0.66–0.83), 0.80 (95% CI 0.74–0.85), 3.80 (95% CI: 2.90–4.80), 0.30 (95% CI 0.22–0.42), and 12 (95% CI 8–19), respectively (Figure 2), and the area under SROC curve was 0.85 (Figure 3).

**FIGURE 4:**
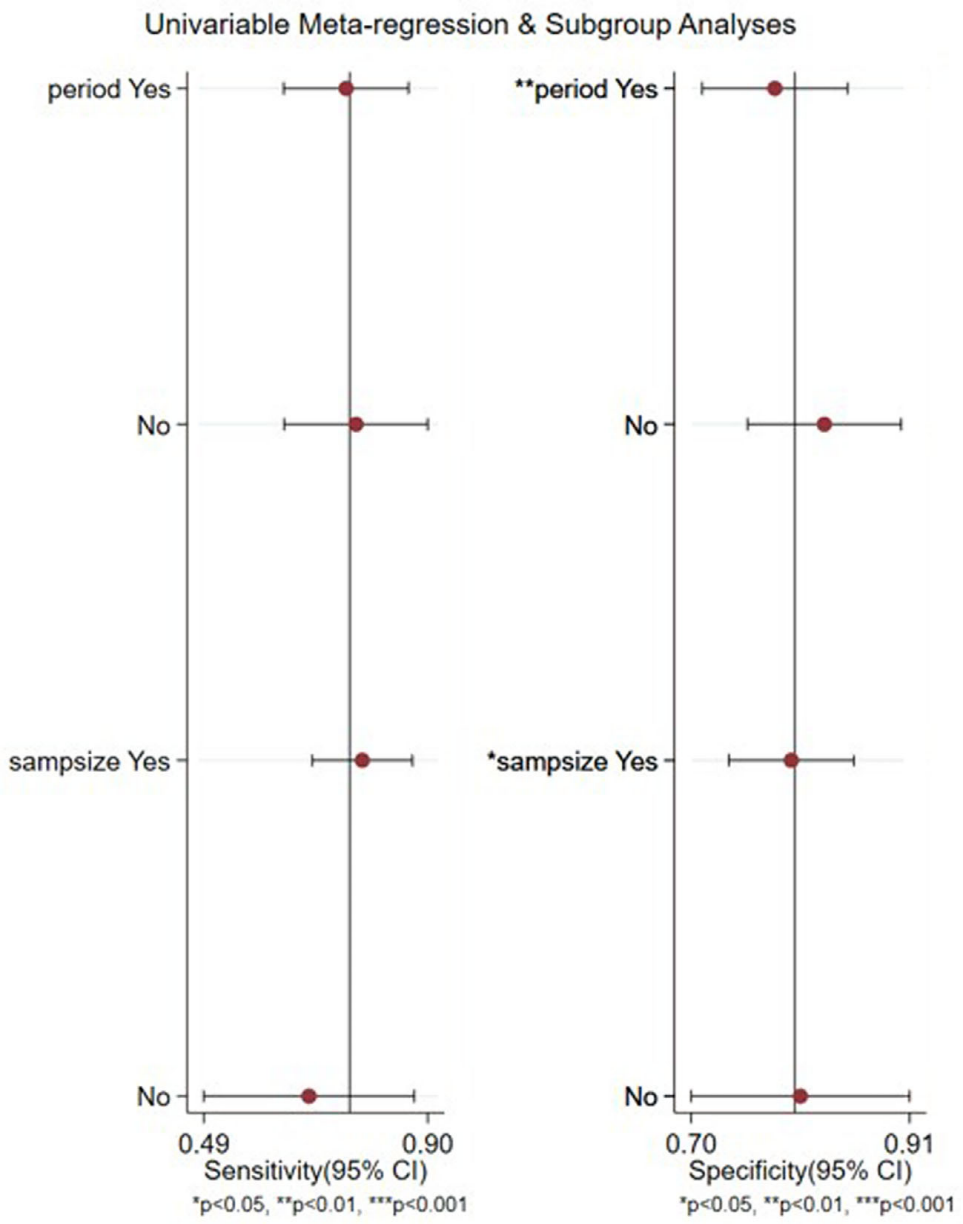
Meta regression analyses for bone metastases disease detection by serum NTx

Meta regression and subgroup analyses showed that the SROC curve did not show a significant “shoulder arm” distribution, which suggests that the heterogeneity was not caused by a threshold effect. Meta regression analysis (Figure 4) was conducted based on the research years (whether the studies were published after 2012) and sample size (whether more than 100 cases were included). The results showed a significant heterogeneity between the research years and sample size, and subgroup analysis was conducted (Table 3). The findings revealed the lower SPN of serum NTx in diagnosis of bone metastasis of lung cancer patients in the past 10 years compared with that 10 years ago. Studies with a sample size of 100 cases or more had a higher diagnosis of bone metastasis in lung cancer patients than those with a sample size of fewer than 100 cases.

**TABLE 3:** Subgroup analysis of NTx

Subgroup		*n*	Pooled sensitivity (95% CI)	Pooled specificity (95% CI)
Total		14	0.76 (0.66–0.83)	0.80 (0.74–0.85)
Period	Yes	8	0.75 (0.64–0.86)	0.78 (0.71–0.85)
	No	6	0.77 (0.64–0.90)	0.83 (0.75–0.91)
Sample size	Yes	10	0.78 (0.69–0.87)	0.80 (0.73–0.86)
	No	4	0.68 (0.49–0.87)	0.80 (0.70–0.91)

**FIGURE 5:**
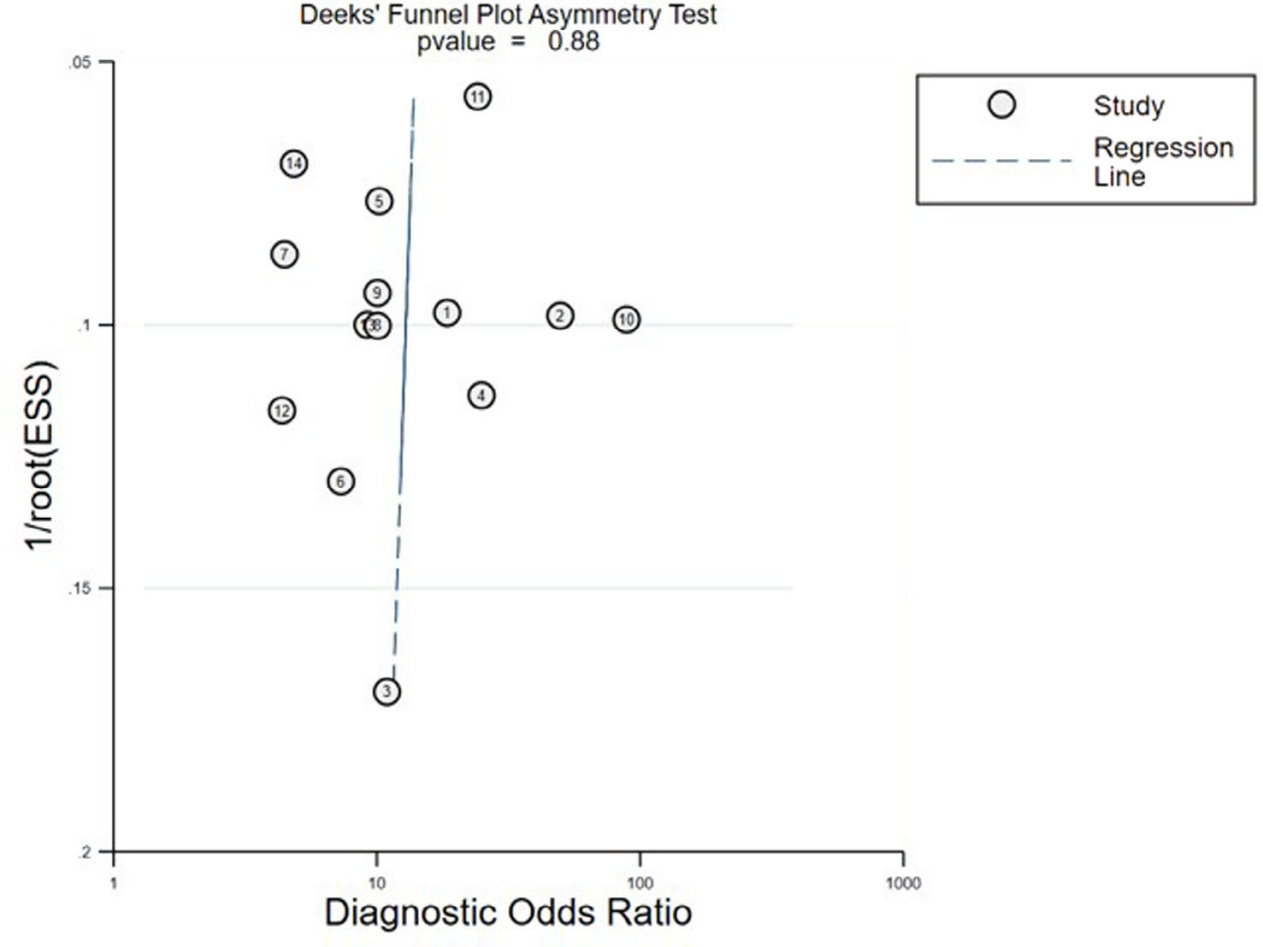
Publication bias for bone metastases disease detection by serum NTx

For publication bias, the linear regression method was used to detect the asymmetry of funnel plots. The results showed no significant asymmetry in the funnel plots (*p* = 0.88), and the regression line in Deek's funnel plots was vertical, which indicates the low possibility of publication bias (Figure 5).

## DISCUSSION

Lung cancer is a malignant tumor with high incidence rate and mortality, and patients are usually in the terminal stage when diagnosed with this condition. Bone is a common metastatic site of lung cancer. Bone pain and complications caused by bone metastasis greatly reduce the quality of life and survival time of patients, therefore the detection, diagnosis, and treatment for bone metastasis is significant for lung cancer patients. Imaging methods, including X‐ray, computed tomography (CT), magnetic resonance imaging (MRI), positron emission tomography (PET)‐CT, and radionuclide bone imaging, are commonly used to detect bone metastasis of malignant tumors. However, these methods have limitations in the detection of early bone metastasis given that this condition often occurs when bone loss is less than 50%. Moreover, CT, MRI, and PET‐CT are expensive and pose radiation risks. Therefore, bone metabolic markers are significant for the diagnosis of early bone metastasis.

NTx is a marker of bone metabolism generated by collagen fiber degradation during osteoclast bone absorption. This marker is an important indicator that reflects the absorption of bone matrix and is used to evaluate osteoclast activity. Compared with other markers of bone metabolism, NTx has a better diagnostic accuracy.^22^ A previous meta‐analysis performed by Liu et al.^23^ showed that the increased level of NTx was closely related to bone metastasis of malignant tumors. In addition, several studies have shown that the level of urinary NTx significantly increased in untreated patients with bone metastasis, and this finding was related to the degree and type of bone metastasis; the increase in urinary NTx levels increases the risk of SREs and death in patients with bone metastasis.^24^, ^25^ However, a limited amount of research has focused on serum‐NTx and bone metastasis of lung cancer patients, and their sample size was small, therefore this study analyzed the clinical application value of serum NTx in the diagnosis of bone metastasis in lung cancer patients through quantitative and systematic evaluation.

In Liu's meta‐analysis,^23^ 11 original studies were included and the authors found that the overall sensitivity and specificity of serum NTx (sNTx) for discerning bone metastasis were 0.74 (95% CI 0.67 to 0.79) and 0.85 (95% CI 0.80 to 0.89), respectively. The area under the SROC curve was 0.8889 (SE = 0.0255) for sNTx.

**FIGURE 6:**
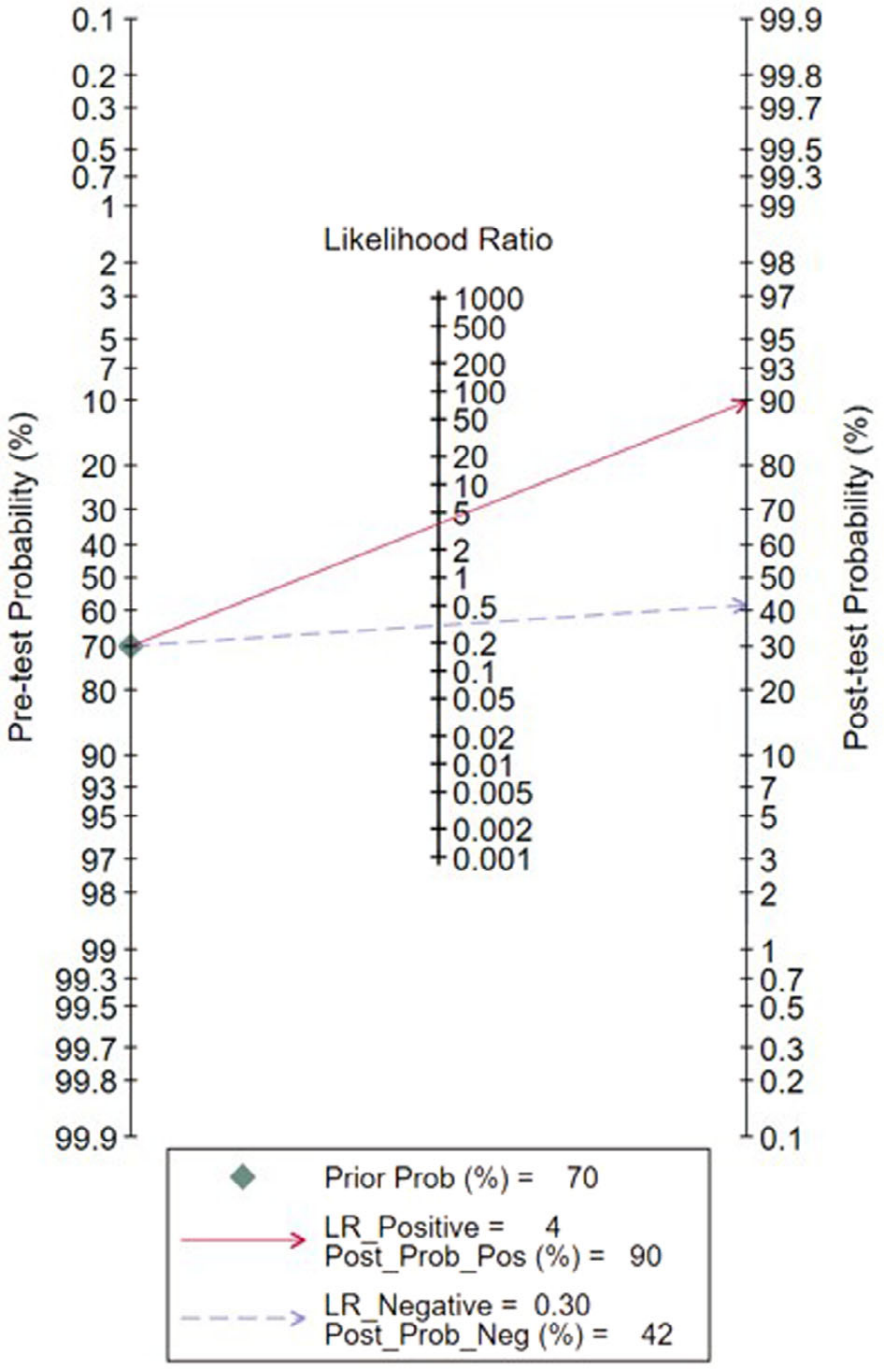
Fagan nomogram for bone metastases disease detection by serum NTx

This study conducted an updated meta‐analysis of 14 diagnostic studies on serum NTx in the diagnosis of bone metastasis in lung cancer patients at home and abroad. Compared to Liu's work, three recently published studies relevant to serum cross‐linked N‐telopeptide of type I collagen as a potential diagnostic marker for bone metastasis in lung cancer were further included and made analysis with a total of 1742 patients of lung cancer included. The cut‐off values for the serum NTx used in various studies were inconsistent, but the results had high SEN and SPN. The meta‐analysis results showed that the SEN and SPN of serum NTx in the diagnosis of lung cancer bone metastasis were 76% and 80%, respectively, and the area under the SROC curve was 0.85. The SPN was higher than the SEN, but the overall diagnostic value was high. In addition, the use of serum NTx was promoted in clinical practice. The area under the curve had a low diagnostic value between 0.5 and 0.7, a good diagnostic value between 0.7 and 0.9, and a high diagnostic value above 0.9. In addition, serum sample collection was more convenient and flexible than imaging examination. The Fagan diagram in Figure 6 shows that when the pretest probability of bone metastasis in lung cancer patients is 70% by imaging inspection, and the results of serum NTx detection are consistent, the post‐test probability can be increased to 90%; otherwise, the post‐test probability is 42%, which suggests that the combination of serum NTx and imaging inspection can accurately determine the degree of bone metastasis in lung cancer patients.

The articles included in this study were high quality and the Deek's funnel chart indicated no evident publication bias. Thus, the analysis results of this study are stable and reliable but also have shortcomings. First, several studies^26^ have revealed that serum NTx has a high value in the diagnosis of bone metastasis in non‐small‐cell lung cancer. However, most of the articles included in this study cannot be used to extract detailed information. Thus, no further analysis was conducted. In the future, more prospective randomized controlled clinical studies are needed to determine the accuracy of serum NTx in the diagnosis of bone metastasis in lung cancer under various circumstances. Second, numerous outcome indicators in this study presented evident heterogeneity, which may be related to the different cut‐off values of serum NTx used in the included research. In addition, this study only considered Chinese and English articles, and the number of english articles in the included literatures was small. Thus, a large language bias possibly exists.

In consideration of different research methods, the accuracy of serum NTx in independent diagnosis of bone metastasis in lung cancer cannot be proven at present. However, the diagnostic value of serum NTx for bone metastasis in lung cancer patients cannot be ignored. Imaging examination combined with serum NTx detection can be used in clinical work to diagnose bone metastasis in lung cancer patients and provide a basis for the rational selection of treatment plans.

## CONFLICT OF INTEREST

The authors have no conflict of interest related to this work.
